# Subcutaneous injection of genetically engineered exosomes for androgenic alopecia treatment

**DOI:** 10.3389/fbioe.2025.1614090

**Published:** 2025-05-30

**Authors:** Yukun Liu, Yonghong Liu, Jing Zhao, Tianyi Deng, Yinyin Ben, Ruitao Lu, Xusha Zhou, Runbin Yan, Xiaoqing Chen, Jian V. Zhang, Grace Zhou

**Affiliations:** ^1^ Shenzhen Key Laboratory of Metabolic Health, Center for Energy Metabolism and Reproduction, Shenzhen Institutes of Advanced Technology, Chinese Academy of Sciences, Shenzhen, China; ^2^ Shenzhen International Institute for Biomedical Research, Shenzhen, China; ^3^ ImmVira Co., Ltd., Shenzhen, China; ^4^ Faculty of Pharmaceutical Sciences, Shenzhen University of Advanced Technology, Shenzhen, China; ^5^ Sino-European Center of Biomedicine and Health, Shenzhen, China

**Keywords:** EX104, engineered exosomes, androgenetic alopecia, hair regrowth, alopecia

## Abstract

Androgenetic alopecia (AGA) is a common disorder that negatively impacts quality of life but remains challenging to treat effectively. The hair loss observed in AGA is the consequence of a gradual reduction in the duration of the anagen phase concomitant of the miniaturization of the hair follicles and subsequent atrophy. This process of miniaturization is associated with abnormalities in the Dihydrotestosterone (DHT) induced dermal papilla cells (DPCs). DHT induces DPCs senescence and promotes apoptosis of vascular endothelial cells and keratinocytes via the DPCs paracrine pathway, which ultimately leads to follicular miniaturization. In this study, we developed a multifunctional exosome-based targeted delivery platform, designated as EX104, through the engineering of HEK-293 cells to express a combination of therapeutical molecules, including WNT10B, VEGFA, and FGF7. EX104 reversed the hair follicle miniaturization phenotype in DHT-induced DPCs. Furthermore, it demonstrated significant hair growth-promoting effects in the murine model of androgenetic alopecia by remodeling the follicular microenvironment and restoring miniaturized hair follicles. Topical EX104 application demonstrated comparable hair growth promotion to first-line minoxidil, while significantly outperforming it in stimulating capillary growth and follicular proliferation. EX104 represents a promising and innovative strategy for AGA management and follicular regenerative therapy.

## 1 Introduction

Androgenetic alopecia (AGA), the most common form of hair loss worldwide, does not directly compromise physical health but profoundly affects psychological wellbeing and quality of life (Russo et al., 2018; Lee et al., 2012; Wang et al., 2010). The pathogenesis of AGA is multifactorial, involving complex genetic predispositions and environmental interactions. Notably, it has been demonstrated that androgen receptor expression is increased in dermal papilla cells (DPCs) in the scalp of patients with AGA, which results in heightened sensitivity to androgens (Inui and Itami, 2011). Androgens, such as dihydrotestosterone (DHT), exert their effects on DPCs, leading to the loss of their replication potential and the capacity to regulate the hair follicle cycle. Concurrently, DPCs secrete negative regulators (e.g., DKK1, TGFβ1, IL-6) (Inui and Itami, 2011; Premanand and Reena Rajkumari, 2018), which induce keratinocyte and vascular endothelial cell apoptosis, triggering microvascular atrophy, inflammation, follicular structural damage, and progressive hair follicle miniaturization (Jung et al., 2022).

The complexity of AGA poses significant therapeutic challenges. Currently, only two U.S. Food and Drug Administration (FDA) approved treatments exist, including topical minoxidil and oral finasteride (Vasserot et al., 2019). Topical minoxidil has been demonstrated to promote hair growth; however, the potential for irritant dermatitis to occur during treatment must be acknowledged. Furthermore, temporary shedding may manifest at the outset of treatment. And some patients do not respond to minoxidil (Gupta et al., 2021). While finasteride is effective for androgenetic alopecia (AGA), its long-term use is associated with a low risk of persistent adverse effects, including sexual dysfunction (erectile dysfunction, reduced libido) and neuropsychiatric symptoms (depression, anxiety) (Traish, 2020). Notably, finasteride is contraindicated in pregnancy per FDA guidelines; therefore, females of reproductive potential must use effective contraception during treatment (Starace et al., 2019). Therefore, the search for a method that inhibits hair loss and promotes hair growth with few side effects is a pressing need.

The progression of androgenetic alopecia is associated with disruption of the follicular microenvironment, with loss of function and apoptosis occurring within the hair follicle in the dermal papilla cells (Li et al., 2023), vascular endothelial cells (Deng et al., 2022), and keratinocytes (Kwack et al., 2008), which play a pivotal role in disease progression. The combined effect of these cellular dysregulations is the promotion of dermal vascular atrophy, nutrient deprivation and follicular miniaturization, which become increasingly pronounced with the progression of the disease. It has been demonstrated that VEGFA (Li et al., 2012; Ding et al., 2024), WNT10B (Tosti et al., 2016) and FGF7 (Gentile and Garcovich, 2019) regulate hair follicle development and the hair cycle through the involvement of hair papilla cells, vascular endothelial cells and keratinocytes, thereby promoting hair growth.

Exosomes, naturally occurring nanovesicles (40–160 nm in diameter), have emerged as promising drug delivery vehicles. Secreted by all cell types, these endogenous extracellular vesicles mediate intercellular communication through cargo transfer, including DNA, RNA, lipids, and proteins (Kalluri and LeBleu, 2020). Their biocompatibility, low immunogenicity, and structural stability make them ideal for targeted therapeutic delivery (Kimiz-Gebologlu and Oncel, 2022). Therefore, exosomes can be loaded with specific proteins or effectors and deliver with remarkable stability (Liang et al., 2021).

Here, we report the development of EX104, an engineered exosome therapeutic encapsulating a synergistic combination of VEGFA, WNT10B, and FGF7 to treat AGA. The therapeutic profile of EX104 was comprehensively assessed through a translational research framework, including *in vitro* DHT-challenged DPCs functional assays and *in vivo* DHT-mediated AGA mouse models. Collectively, our data demonstrates that EX104 represents a promising and innovative strategy for AGA management and follicular regenerative therapy.

## 2 Results

### 2.1 Development and characterization of EX104

**FIGURE 1:**
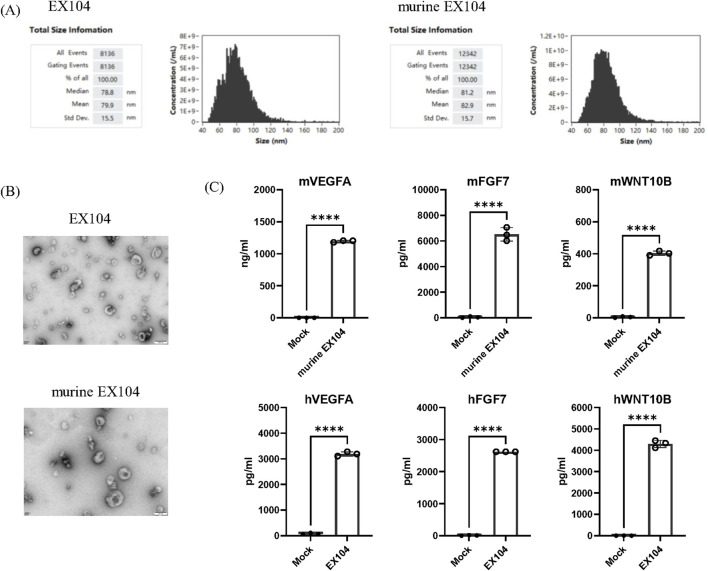
Characterization of EX104. Representative size distribution **(A)** of EX104 and murine EX104. **(B)** TEM images of EX104 and murine EX104 engineered exosomes. **(C)** Protein level of VEGFA, FGF7 and WNT10B by ELISA from Mock (293-exo), EX104 and murine EX104 (1E+12 particles/mL). Data are shown as mean ± standard deviation (SD). Statistical analysis was performed by Student’s *t*-test. Significance level: ****P < 0.0001.

The engineered cell line producing engineered exosome was developed by infection with lentivirus containing three cargo genes (WNT10B, FGF7, and VEGFA) in HEK-293 cells. Subsequently, the engineered exosomes were isolated from culture conditioned medium of the stable cell line by ultracentrifuge. Two engineered exosome variants were successfully constructed for this study: EX104 and murine EX104. EX104 was loaded with human FGF7, WNT10B, and VEGFA, while murine EX104 was loaded with their murine homologues. Mock, as control engineered exosome, from HEK-293 by infection with lentivirus containing empty backbones. The engineered exosomes were isolated and their size distribution, as well as the effectors expression (FGF7, WNT10B, and VEGFA) were characterized. The average size distribution of EX104 and murine EX104 was determined to be predominantly around 80 nm in diameter by nano-flow cytometry (NanoFCM) (Figure 1A). TEM analysis of EX104 and murine EX104 revealed characteristic cup-shaped vesicles with intact bilayer membranes, consistent with canonical exosome morphology (Figure 1B). Subsequently, ELISA demonstrated a significant increase in the levels of FGF7, WNT10B, and VEGFA in EX104 and murine EX104 in comparison to mock (Figure 1C). The data substantiates the successful preparation of the engineered exosomes EX104 and murine EX104.

### 2.2 Effects of EX104 on the viability of DPCs with or without DHT-induced

**FIGURE 2:**
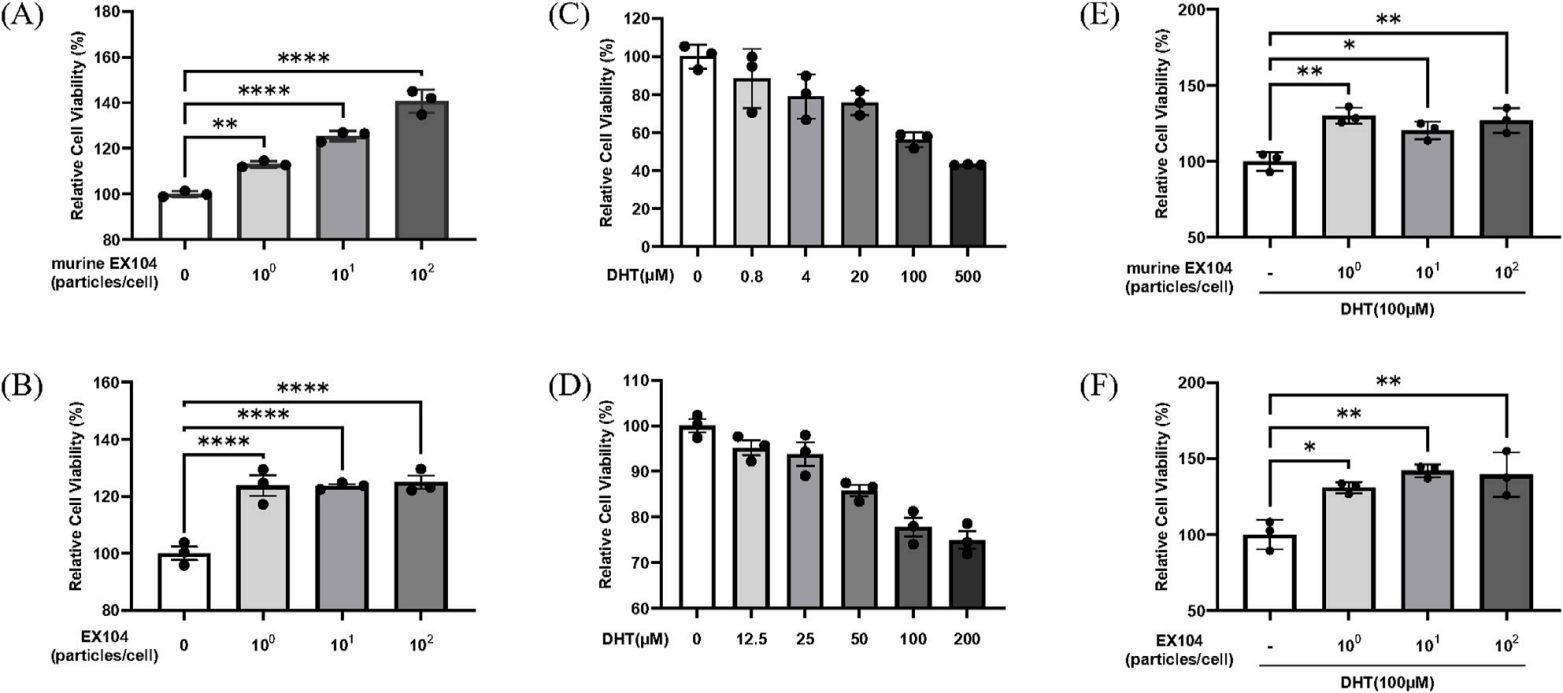
Effects of EX104 on the viability of DPCs with or without DHT-induced. Effects of different concentrations of EX104 alone on viability of mDPCs **(A)** and hDPCs **(B)**. Effects of different concentrations of DHT alone on the viability of mDPCs **(C)** and hDPCs **(D)**. mDPCs **(E)** or hDPCs **(F)** were pretreated with DHT (100 μM) for 24 h and subsequently incubated with or without different concentrations of murine EX104 or EX104, respectively. Cell viability was determined using the CellTiter-Glo Luminescent Cell Viability Assay. Statistical analysis was performed by one-way ANOVA. Significance level: *P < 0.05, **P < 0.01, ***P < 0.001, ****P < 0.0001.

It has been reported that DHT can result in a loss of DPCs replication potential (Zhang et al., 2021; Winiarska et al., 2006). Our findings revealed that DHT treatment demonstrated an inhibitory effect on DPCs proliferation in a dose-dependent manner (Figures 2C,D). Accordingly, a DHT concentration of 100 μM was selected to mimic the pathological levels observed in AGA patients. The results demonstrate that EX104 has the capacity to markedly enhance human dermal papilla cells (hDPCs) proliferation, both in the presence and absence of DHT (Figures 2B,F). Furthermore, murine EX104 exhibited comparable outcomes on murine dermal papilla cells (mDPCs) (Figures 2A,E). The data suggests that EX104 has a promoting effect on DPCs proliferation, notably, a reversing effect on the DHT-induced loss of DPCs replication potential.

### 2.3 EX104 reversed the hair follicle miniaturization phenotype in DHT-induced DPCs

**FIGURE 3:**
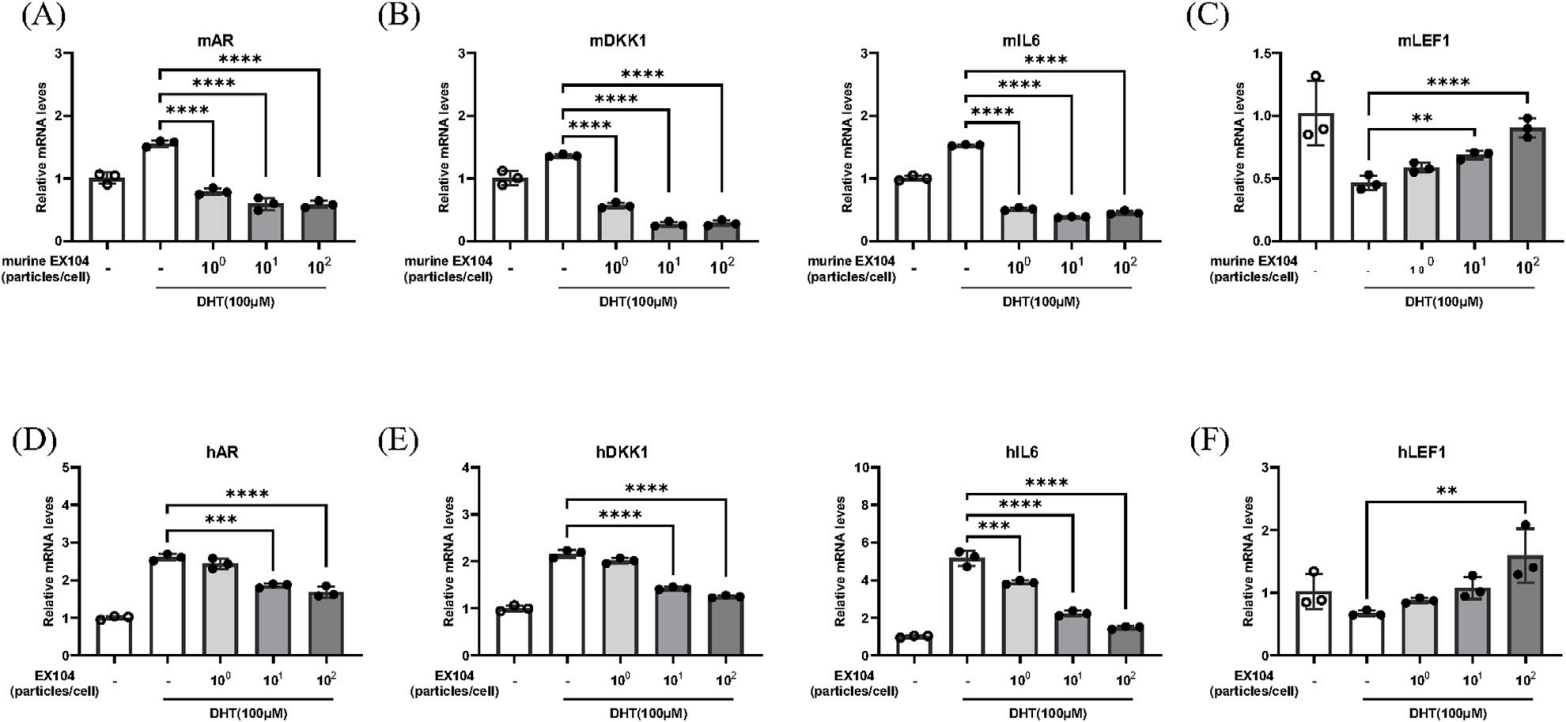
Effects of EX104 on hair follicle miniaturization phenotype of DHT-induced DPCs. mDPCs **(A–C)** and hDPCs **(D–F)** were pretreated with DHT (100 μM) for 24 h and subsequently incubated with or without different concentrations of murine EX104 and EX104 for 24 h, respectively. Whole cells were then collected for the purpose of determining the gene expression of DKK1, AR, IL-6 and LEF1 using real-time RT-PCR. Statistical analysis was performed by one-way ANOVA. Significance level: *P < 0.05, **P < 0.01, ***P < 0.001, ****P < 0.0001.

The binding of androgens to the androgen receptor (AR) induces the nuclear translocation of the AR, which then induces AR expression and acts as a transcription factor for androgen-responsive genes (Inui and Itami, 2011). The objective was to ascertain whether EX104 affects DHT-induced AR expression by measuring AR mRNA expression in DPCs. The results demonstrated that DHT increased AR mRNA expression, however, this was reversed in both hDPCs and mDPCs when treated with EX104 and murine EX104, respectively (Figures 3A,D). In particular, the treatment of EX104 resulted in a reduction of AR mRNA expression to levels comparable to those observed in the control group in mDPCs.

In patients with androgenetic alopecia, the level of AR mRNA in DPCs is higher than in normal people, which results in sustained binding of DHT to the AR of DPCs. This results in the DPCs exhibiting a follicular miniaturization phenotype, including loss of replicative potential, reduction of molecular markers and secretion of inhibitory factors, such as DKK1 and IL6 (Premanand and Reena Rajkumari, 2018). DKK1 functions as a negative regulator of the WNT signaling pathway, whereas IL-6 is an inflammatory cytokine. These processes inhibit the transition of hair follicles from the telogen to anagen phase. The mRNA levels of DKK1 and IL6, which are known to promote hair follicle regression, were detected. As anticipated, the elevated levels of DKK1 and IL6 mRNA were effectively reversed by EX104 treatment (Figures 3B,E).

The morphogenesis and growth cycle of hair follicles is regulated by a number of signaling pathways, including the WNT pathway. LEF1 (Wang et al., 2012), which is essential for the WNT signaling pathway, is highly expressed during the anagen phase of hair follicles and can regulate hair growth by promoting β-catenin translocation to the nucleus. The mRNA expression of LEF1 was subsequently detected. As anticipated, the reduction in LEF1 mRNA levels observed with DHT was reversed by EX104 treatment (Figures 3C,F).

In conclusion, the results demonstrated that EX104 treatment effectively reversed the follicular miniaturization phenotype of DPCs induced by DHT, thereby facilitating the activation and maintenance of the hair cycle.

### 2.4 EX104 promotes hair growth in androgenetic alopecia mice

**FIGURE 4:**
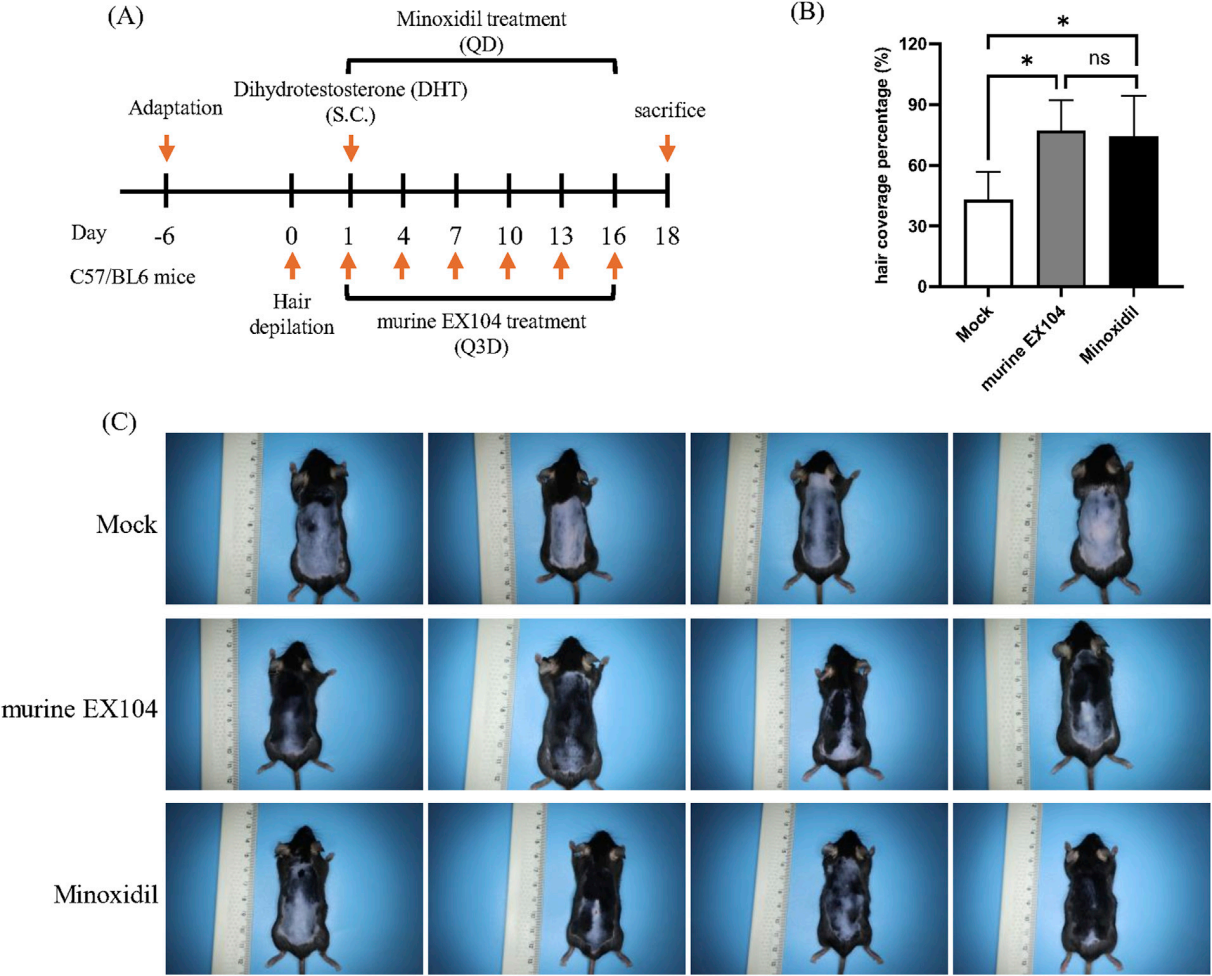
Therapeutic efficacy of murine EX104 in DHT-induced AGA C57BL/6 mouse models. **(A)** Animal experimental schedule for inducing androgenetic alopecia mice model. DHT was hypodermic injection, and murine EX104 was injected subcutaneously once every 3 days, Minoxidil was topically administered once every day. **(B)** Hair coverage percntage (%) on the dorsal skin for each group on days 15 after depilation. **(C)** Each photograph documents the hair regrowth state of individual mouse on day 15 post-administration. Statistical analysis was performed by one-way ANOVA. Significance level: *P < 0.05, **P < 0.01, ***P < 0.001, ****P < 0.0001.

Based on the studies above, we proceeded to assess the hair-growth-promoting efficacy of murine EX104 in a DHT-induced AGA mouse model. C57BL/6 mice were divided into three groups. Hair was removed by using depilatory cream to observe the pink skin. Subsequently, DHT (30 mg/kg) was injected subcutaneously to establish the AGA mouse model. The three AGA mouse model groups were treated separately for 18 days as follows: Mock (subcutaneous injection, 0.1 mL/animal, every 3 days); murine EX104 (subcutaneous injection, 0.1 mL/animal, every 3 days); minoxidil (topical application, 0.2 mL/animal, daily). As illustrated in the figure, at day 15 post-treatment initiation, mice in the murine EX104 treatment group exhibited greater than 70% hair coverage, in comparison to 40% observed in the control group (Figures 4B,C). The data indicated that hair coverage in the murine EX104-treated group exhibited a statistically significant increase compared to the control group. This suggests that murine EX104 plays a pivotal role in promoting hair regrowth in androgenetic alopecia mice.

### 2.5 EX104 inhibits inflammation and enhances Wnt/β-catenin signaling in hair follicles

**FIGURE 5:**
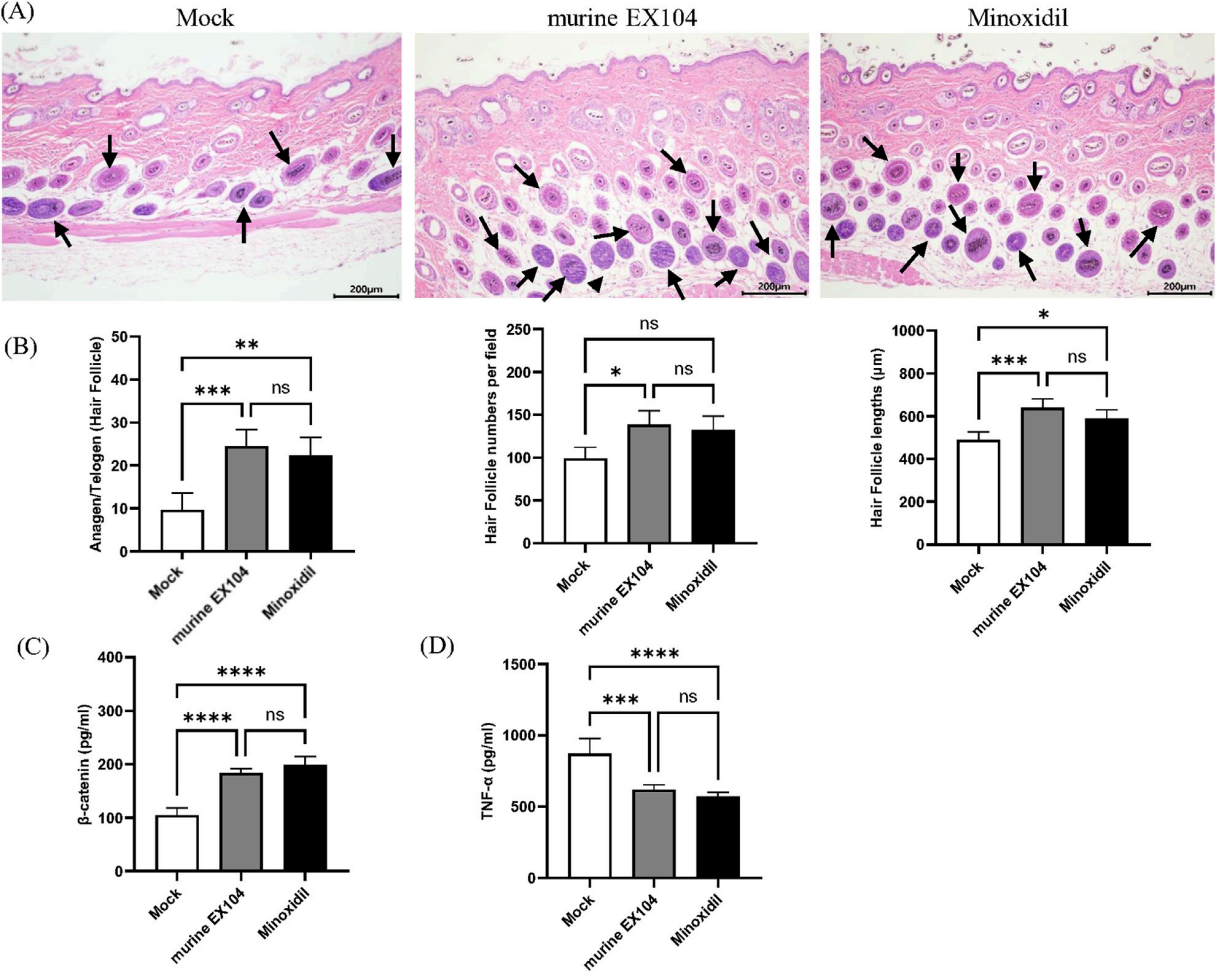
Effects of murine EX104 on hair follicles and inflammation in DHT-induced AGA C57BL/6 mouse models. **(A)** Depilated mice skin tissue was paraffin sectioned and histologic examination was performed by HE staining. Images were captured by digital slide scanner. *N* = 6. Scale bars are 200 μm. **(B)** Hair follicle number, Hair follicle lengths and Anagen/Telogen hair follicle (%) was measured in the same random area. Quantitative Analysis of β-Catenin **(C)** and TNF-α **(D)** levels in mouse skin tissues was performed by ELISA. Statistical analysis was performed by one-way ANOVA. Significance level: *P < 0.05, **P < 0.01, ***P < 0.001, ****P < 0.0001.

To determine the state of the hair follicle, dorsal skin samples were taken from all subjects and studied using H & E staining (Figure 5A). The number of hair follicles per field was counted quantitatively, and the morphology of the inner and outer root sheath of hair was displayed. Hair follicle numbers and hair bulb size could be modulated by AGA pathogenesis, and we observed the effect of murine EX104 on them. We found that treatment with murine EX104 significantly increased the number and diameter of hair follicles in androgenetic alopecia mice (Figure 5B). This was comparable to the effect of minoxidil. In addition, treatment with murine EX104 significantly increased the anagen-to-telogen ratio in androgenetic alopecia mice (Figure 5B).

Inflammation promotes anagen to telogen transition and has been associated with the progression of alopecia. We detected the level of TNF-α associated with androgenetic alopecia in the dorsal skin of androgenetic alopecia mice. Treatment with murine EX104 significantly reduced the level of TNF-α in the dorsal skin of androgenetic alopecia mice (Figure 5C). β-catenin, a component of the WNT signaling pathway, plays a pivotal role in the development and growth of hair follicles. Treatment with murine EX104 significantly increased the level of β-catenin in the dorsal skin of androgenetic alopecia mice (Figure 5D). The data demonstrates that murine EX104 has the capacity to enhance the microenvironment of the hair follicle in androgenetic alopecia mice, exhibiting the potential to promote hair growth.

### 2.6 EX104 promoted hair follicles proliferation and enhanced angiogenesis

In general, hair follicles are surrounded by deep dermal vascular plexuses. The associated blood vessels function to supply nutrients to the growth and development of hair follicle. Proper blood supply is necessary for effective hair follicle growth (Bassino et al., 2015). However, blood vessels regress in hair follicle of balding scalps, which may contribute to hair follicle miniaturization in androgenetic alopecia patients (Deng et al., 2022). To evaluate the histological changes in androgenetic alopecia mice following murine EX104 treatment, skin samples were taken from all subjects and studied through immunohistochemistry for the proliferation marker Ki67 and the angiogenesis marker CD31.

**FIGURE 6:**
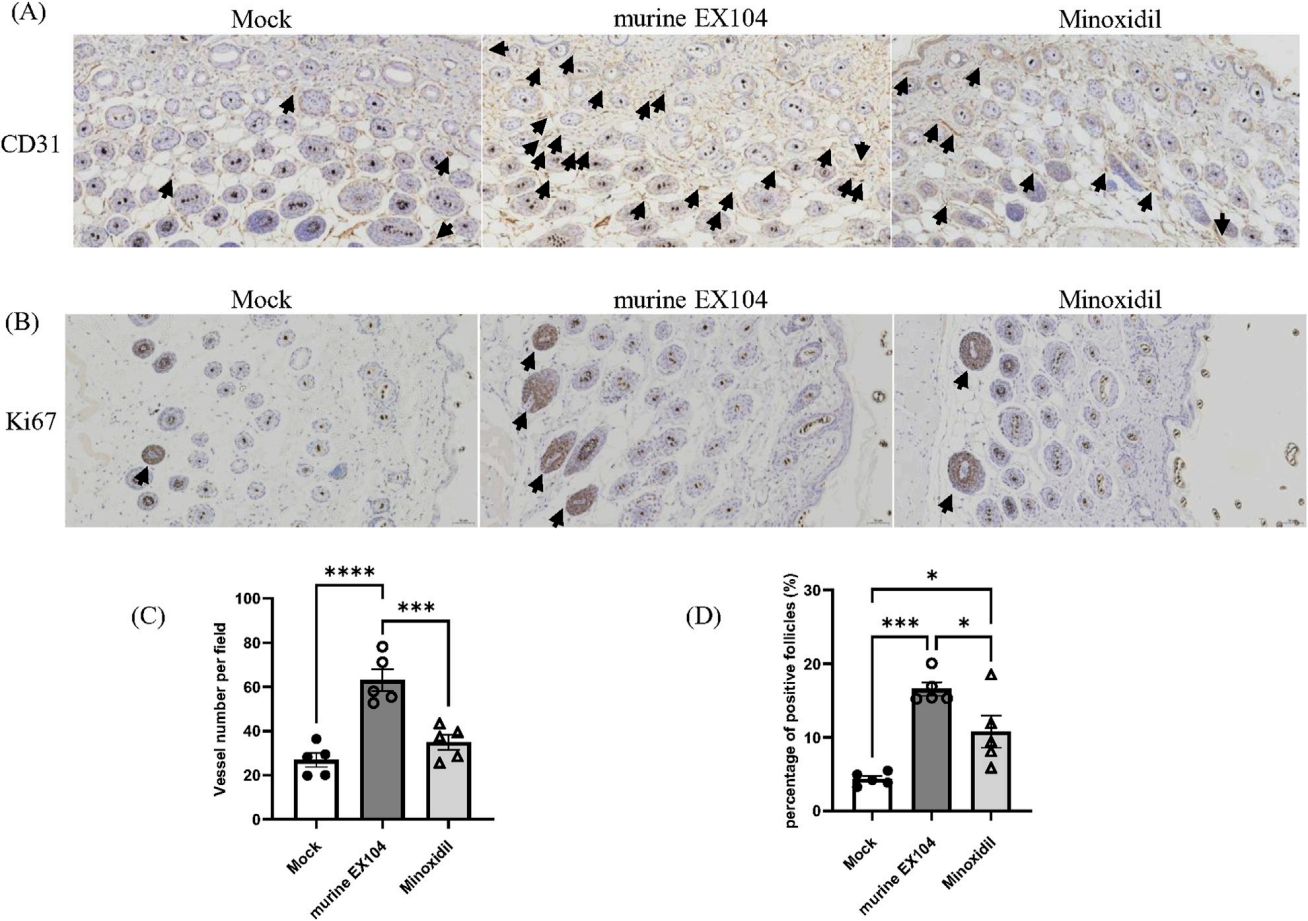
Effects of murine EX104 on blood vessels and proliferating hair follicles in DHT-induced AGA C57BL/6 mouse models. **(A)** CD31 immunohistochemical staining of dermal sections with 20 × objective lens (black arrows indicate CD31-positive micro-vessels). Scale bar = 50 μm. **(B)** Ki67 immunohistochemically staining of dermal sections with 20 × objective lens (black arrows indicate Ki67-positive hair follicles). Scale bar = 50 μm. **(C)** Quantitative analysis of CD31 staining surrounding hair follicles. **(D)** Quantitative analysis of Ki67 staining inside hair follicles. Statistical analysis was performed by one-way ANOVA. Significance level: *P < 0.05, **P < 0.01, ***P < 0.001, ****P < 0.0001.

The immunohistochemical stain of CD31 showed that the number of blood vessels per field surrounding dermal papillae was significantly increased in the murine EX104 treatment group and Minoxidil treatment group compared to Mock group (Figure 6A). Specifically, the number of blood vessels in murine EX104 treatment group was higher than the Minoxidil group (Figure 6C). The immunohistochemical stain of Ki67 showed that the number of Ki67-positive hair follicles per field was significantly increased in the murine EX104 treatment group and Minoxidil treatment group compared to Mock group (Figure 6B). And the number of Ki67-positive hair follicles in murine EX104 treatment group was higher than the Minoxidil group (Figure 6D). This indicates that murine EX104 could induce anagen phase from telogen phase.

## 3 Discussion

Despite its high prevalence, the precise pathogenic mechanisms underlying AGA remain incompletely understood. Current first-line therapies finasteride (a selective type II 5α-reductase inhibitor) and minoxidil (a potassium channel activator) demonstrate limited therapeutic efficacy, highlighting an unmet clinical need (Devjani et al., 2023). Finasteride, while effective in arresting androgen-mediated follicular damage, may be accompanied by certain adverse effects (Gupta et al., 2022). Minoxidil demonstrates suboptimal efficacy in certain patients, likely attributable to deficient sulfotransferase activity–an essential enzyme required for its metabolic activation into the therapeutically active metabolite, minoxidil sulfate (Suchonwanit et al., 2019). The psychosocial burden of AGA is substantial, particularly as its onset increasingly affects younger populations. This demographic shift, coupled with the limitations of existing therapies, has intensified the demand for novel treatment strategies (Aukerman and Jafferany, 2022). In order to develop new treatments for this condition, it is essential to consider the hair follicle microenvironment and the follicle miniaturization phenotype.

The hair follicle is a complex small organ in the skin in which keratinocytes undergo programmed cell death to form fibers that are characterized by their extremely high tensile strength. Central to its regulatory architecture are DPCs, which serve as mesenchymal signaling hubs governing follicular dimensions (size, diameter, length) and cycling dynamics, particularly anagen phase duration (Chi et al., 2013). Androgenetic alopecia (AGA) is a multifaceted pathological process involving multiple factors. The pathogenic cascade involves DPCs dysfunction (Inui and Itami, 2011): Androgen-mediated induction of miniaturization phenotype, marked by proliferative arrest and functional impairment, Paracrine dysregulation (Kwack et al., 2008; Deng et al., 2022): Aberrant cytokine signaling from DPCs triggers keratinocyte and vascular endothelial apoptosis, Structural collapse: Consequent microvascular degeneration and follicular matrix disruption lead to cycle arrest and irreversible hair loss.

Recent advances in regenerative therapies, including stem cells and platelet-rich plasma, have revealed that a range of cytokines can promote hair follicle development for hair growth, paving the way for new therapies for hair loss (Saceda-Corralo et al., 2023). For example, WNT10B (Lim and Nusse, 2012) has been shown to stimulate DPCs proliferation and activate follicular stem cell niches, while VEGFA (Li et al., 2012) has been shown to promote neovascularization and enhance DPCs mitotic activity. Also, FGF7 (Gentile and Garcovich, 2019) has been shown to expand follicular dimensions and prolong anagen phase. Building on these insights, we developed EX104 as a targeted delivery platform co-encapsulating WNT10B, VEGFA, and FGF7. This multifunctional system aims to remodel the pathological follicular microenvironment, offering a novel therapeutic approach for AGA management.

In this study, we demonstrated that EX104 treatment significantly enhanced DPCs proliferation. *In vitro* analysis revealed significant pro-proliferation effects of EX104 treatment on both human dermal papilla cells (hDPCs) and murine dermal papilla cells (mDPCs). *In vivo* studies demonstrated that EX104 treatment significantly increased the number of Ki67-positive hair follicles in the skin of AGA mice. These findings indicate that EX104 can directly reverse the inhibition of DPCs proliferation by DHT. Secondly, EX104 treatment was found to significantly promote the number of blood vessels in the skin of AGA mice. This angiogenic response could restore the nutrient supply to the hair follicle microenvironment and induce hair growth.

In addition, the therapeutic potential of EX104 treatment extends to reversing the DHT-induced miniaturization phenotype in DPCs. Mechanistically, sustained DHT exposure induces DPC follicle miniaturization, triggering cytokine secretion (e.g., elevated AR, DKK1, IL-6) that exacerbates disease progression. Specifically, DKK1 (Kwack et al., 2008) promotes follicular keratinocyte death and structural damage, while IL-6 (Kwack et al., 2012) induces microinflammation, and collectively, they advance AGA pathogenesis. *In vitro* studies have shown that EX104 treatment downregulates DHT-induced expression of AR, DKK1 and IL-6, and promotes expression of the pro-hair growth indicators Lef1. *In vivo* studies have demonstrated that EX104 treatment significantly augmented the content of β-catenin and diminished the content of TNF-α in the skin of AGA mice. Collectively, these multimodal effects of EX104 treatment enable reprogramming of the perifollicular niche, reactivating hair follicle regeneration through reprogrammed DPCs, restoration of WNT/β-catenin signaling axis and concurrent suppression of inflammatory and apoptotic pathways.

Engineered exosomes, as advanced therapeutic delivery platforms, have emerged as promising next-generation drug delivery vehicles, offering distinct advantages over synthetic systems such as liposomes and polymeric nanoparticles. They have lower immunogenicity, toxicity, higher stability, and internal routing (Kimiz-Gebologlu and Oncel, 2022). The development of engineered exosome technology has further addressed limitations associated with natural exosome, enabling standardized production protocols and Precise loading of therapeutic cargo. We developed EX104 as a multifunctional delivery platform and encapsulated three synergistic bioactive factors (WNT10B, VEGFA, FGF7). These attributes position EX104 as a clinically translatable therapeutic strategy for AGA.

The limitations of our study include reliance solely on mouse models for pharmacodynamic evaluations *in vivo*. Given the structural differences between murine and human hair systems, these models cannot fully recapitulate the pathological process of human AGA. To address this limitation, we developed EX104 loaded with human homologues (FGF7, WNT10B, and VEGFA) and evaluated its efficacy in rescuing the follicular miniaturization phenotype in DHT-induced DPCs. We used a single dose of exosomes in mouse models, determined based on previous experience. Future studies should incorporate dose-response analyses to establish both the minimal effective dose and optimal therapeutic window for EX104. Furthermore, to advance clinical translation, comprehensive evaluation of the safety profile of EX104 remains imperative. Future studies should prioritize systematic preclinical assessments of EX104’s safety and pharmacokinetics, including absorption, distribution, local/systemic toxicity, immune responses, and off-target signaling effects.

## 4 Conclusion

In patients with AGA, sustained DHT exposure drives hair follicle miniaturization. EX104 ameliorates this process by rescuing the follicular miniaturization phenotype of DPCs. Concurrently, it stimulates microvascular angiogenesis and reinforces follicular structural integrity, ultimately facilitating the transition of hair follicles into the anagen growth phase. In summary, the results indicate that EX104 promise as an innovative and effective approach for the treatment of AGA.

## 5 Materials and methods

### 5.1 Generation of engineered exosomes EX104

The amino acid sequences of all target genes, including FGF7, WNT10B, and VEGFA were derived from UniProt and the corresponding DNA sequences were synthesized by General Biotechnology (Chuzhou, China). T2A peptide was inserted between these gene sequences so that the resulting polypeptide could be dissociated into individual therapeutic proteins upon translation. Subsequently, the DNA sequence containing these three genes was cloned into the lentiviral plasmid pCDH-CMV-MCS-EF1a-GFP (System Biosciences). The four plasmids of the third-generation system (pCDH-CMV-FGF7-T2A-VEGFA-T2A-WNT10B-EF1a-GFP, pRSV Rev, pMDLg-pRRE, and PMD2.G) were used for lentivirus production by co-transfecting HEK-293T cells. After medium replacement and incubation for another 72 h, the virus-containing supernatant was collected and centrifuged at 3,000 rpm for 5 min to remove cell debris. The viral titer was determined based on GFP transduction efficiency. Finally, stable cell lines were constructed by infecting HEK-293 cells at an MOI of 0.5.

Specifically, the engineered cell line producing EX104 was developed by infection with lentivirus containing three cargo genes, FGF7, WNT10B, and VEGFA in HEK-293 cells. Forty-eight hours later, engineered cells were selected by the addition of antibiotics blasticidin. Afterwards, a single cell colony with GFP expression was selected and cultured in complete medium. The stable cell line was monitored for the expression of GFP and the corresponding cargo proteins during passaging. Next, the supernant from the above stable cell lines was collected for engineered exosomes isolation. In this study, EX104 (functional proteins in human version) and murine EX104 (functional proteins in mouse version) were used in human and murine experiments, respectively.

### 5.2 Isolation of EX104

EX104 were isolated from culture conditioned medium of the stable cell line. Briefly, the medium from HEK-293 was collected and centrifuged at 300 *g* for 10 min, 2,000 × g for 10 min, and 10,000 × g for 30 min at 4°C to remove dead cells and cellular debris. The supernatant was subsequently filtered through a 0.22-μm filter sterilizer. Ultracentrifugation was performed twice using a 70 Ti rotor at 100,000 × g for 70 min at 4°C, and the pellet was then resuspended in PBS.

### 5.3 Characterization of exosomes

TEM was used to confirm the presence of exosomes. Approximately, 20 µL of exosomes were added separately to copper grids. All excess fluids were removed using filter paper, and the samples were negatively stained with 2% uranyl acetate for 30 s. The grids were rinsed in deionized water and allowed to dry overnight. The samples were then air-dried using an electric incandescent lamp and viewed using an electron microscope (Hitachi, S-3000N).

The particle concentration and size distribution of exosomes from stable cell line were analyzed by the nFCM (NanoFCM Inc., Xiamen, China). The nFCM analysis used two single photon counting avalanche photodiodes (APDs) to detect individual particle side scatter (SSC) and fluorescence simultaneously. Firstly, the exosomes pellet was prepared for analysis. Then, 200 nm PE and AF488 fluorophore-conjugated polystyrene beads were used for particle concentration and Silica Nanosphere Cocktail (NanoFCM Inc., Xiamen, China) for particle size distribution. The detector recorded particles passing by during a 1-min interval in each test. Each sample was diluted to reach a particle count within the optimal range of 3,000–9,000 particles per minute. NanoFCM software (NanoFCM Profession V2.0) was used to convert flow rate and side scattering intensity to vesicle concentration and size. The effectors level of WNT10B, FGF7 and VEGFα in Exosome were measured using ELISA kits according to the manufacturer’s instructions.

### 5.4 Cell culture

The mDPCs (PRI-MOU-00100) and mouse dermal papilla stem cells complete culture medium (PCM-M-100) were purchased from Shanghai Zhong Qiao Xin Zhou Biotechnology Co., Ltd. (China). The hDPCs (CTCC-001-0429) and Dulbecco’s modified Eagle medium/F12 (CTCC-002-004) were purchased from Meisen Chinese Tissue Culture Collections (China). Fetal bovine serum (10,270,106) and Penicillin-Streptomycin (15140-122) were purchased from Gibco (United States). mDPCs were cultured in mouse dermal papilla stem cells complete culture medium containing 10% fetal bovine serum, streptomycin (100 μg/mL), and penicillin (100 units/mL). hDPCs were cultured in Dulbecco’s modified Eagle medium/F12 containing 10% fetal bovine serum, streptomycin (100 μg/mL), and penicillin (100 units/mL).

### 5.5 Cell titer-glo cell viability assay

The mDPCs and hDPCs were first seeded and incubated overnight in 96-well plate at a density of 5 × 10^3^ cells per well. The cells were pre-treated with either normal or DHT medium for 24 h. Different concentrations of Mock, EX104 or murine EX104 were then added to the cells for 48 h treatment. The cell viability was determined by using a CellTiter-Glo luminescent cell viability assay kit (Promega) following the manufacturer’s instruction. The plates were scanned with a luminescence microplate reader. The surviving fraction of drug-treated cells was normalized to untreated control. Experiments were performed in technical triplicates over three biological repeats. Survival and significance were determined by GraphPad Prism software.

### 5.6 Real-time quantitative-PCR (RT-PCR)

RNA was extracted with the RNeasy Mini Kit (Qiagen, 74104), and the cDNA was synthesized with HiScript II Q RT SuperMix for qPCR (+gDNA wiper) (Vazyme, R223-01) as instructed. RT-qPCR was performed with SYBR Green qPCR Master Mix (Universal) (MCE (Med Chem Express), HY-K0501A). The list of primer sequences as shown as follow:

Primer name: mGapdh-F; sequence (5′-3′): TCA​CCA​CCA​TGG​AGA​AGG​C

Primer name: mGapdh-R; sequence (5′-3′): GCT​AAG​CAG​TTG​GTG​GTG​CA

Primer name: mLef1-F; sequence (5′-3′): TGT​TTA​TCC​CAT​CAC​GGG​TGG

Primer name: mLef1-R; sequence (5′-3′): CAT​GGA​AGT​GTC​GCC​TGA​CAG

Primer name: mVegfa-F; sequence (5′-3′): GCA​CAT​AGA​GAG​AAT​GAG​CTT​CC

Primer name: mVegfa-R; sequence (5′-3′): CTC​CGC​TCT​GAA​CAA​GGC​T

Primer name: mDkk1-F; sequence (5′-3′): CTC​ATC​AAT​TCC​AAC​GCG​ATC​A

Primer name: mDkk1-R; sequence (5′-3′): GCC​CTC​ATA​GAG​AAC​TCC​CG

Primer name: mAr-F; sequence (5′-3′): CTG​GGA​AGG​GTC​TAC​CCA​C

Primer name: mAr-R; sequence (5′-3′): GGT​GCT​ATG​TTA​GCG​GCC​TC

Primer name: mIl6-F; sequence (5′-3′): CCA​AGA​GGT​GAG​TGC​TTC​CC

Primer name: mIl6-R; sequence (5′-3′): CTG​TTG​TTC​AGA​CTC​TCT​CCC​T

Primer name: hGAPDH-F; sequence (5′-3′): TGT​TGC​CAT​CAA​TGA​CCC​CTT

Primer name: hGAPDH -R; sequence (5′-3′): CTC​CAC​GAC​GTA​CTC​AGC​G

Primer name: hLEF1-F; sequence (5′-3′): GAA​TTA​GCA​CGG​AAA​GAA​AGA

Primer name: hLEF1 -R; sequence (5′-3′): ACC​TGT​ACC​TGA​TGC​AGA​TT

Primer name: hVEGFA-F; sequence (5′-3′): AGG​GCA​GAA​TCA​TCA​CGA​AGT

Primer name: hVEGFA -R; sequence (5′-3′): AGG​GTC​TCG​ATT​GGA​TGG​CA

Primer name: hDKK1-F; sequence (5′-3′): CCT​TGA​ACT​CGG​TTC​TCA​ATT​CC

Primer name: hDKK1-R; sequence (5′-3′): CAA​TGG​TCT​GGT​ACT​TAT​TCC​CG

Primer name: hAR-F; sequence (5′-3′): CCT​GGC​TTC​CGC​AAC​TTA​CAC

Primer name: hAR -R; sequence (5′-3′): GGA​CTT​GTG​CAT​GCG​GTA​CTC​A

Primer name: hIL6-F; sequence (5′-3′): ACT​CAC​CTC​TTC​AGA​ACG​AAT​TG

Primer name: hIL6-R; sequence (5′-3′): CCA​TCT​TTG​GAA​GGT​TCA​GGT​TG

### 5.7 Animals and experimental design

All experiments were performed using 6-week-old male C57BL/6 mice (body weight 18–22 g). Animals were purchased from Cavens (Changzhou, China). At the end of the experiment, mice were euthanized humanely using CO_2_ overdose, followed by cervical dislocation. All animal experiments were approved by the Institutional Animal Care and Use Committee of SAFE (Shenzhen, China) (IACUC-2023-272).

For *in vivo* therapy experiment, the mice were shaved firstly, then hair was removed by using depilatory cream to observe the pink skin. On the next day of depilation, 30 mg/EA DHT (dihydrotestosterone) suspension was injected subcutaneously into five spots of the dorsal depilation area. Animals were randomly divided into three groups to study hair regrowth. Following hair depilation, the dorsal skin of mice was treated with either Mock (vehicle control), murine EX104, or 5% minoxidil (positive control). Exosome treatment dose: A total of 8.0 × 10^9^ particles in 100 μL PBS was injected subcutaneously into five dorsal skin sites (20 μL per injection site) every 3 days. Minoxidil treatment: topical application of 200 µL daily. The dorsal skin was photographed at 0, 9, 12, and 15 days. ImageJ was used to analyze the depilation area and the hair growth area of the mice. Hair coverage % = (new hair area/hair removal area) * 100%. Observation continued over an 18-day treatment period, then animals were euthanized humanely and the dorsal skin was harvested.

A part of skin samples was fixed in 4% paraformaldehyde and subsequently embedded in paraffin, cut into 5 μm sections, deparaffinized, rehydrated, and then stained with hematoxylin and eosin, followed by dehydration and final sealing. Skin thickness and diameter of hair follicles were measured using Slideviewer software (3DHISTECH, Budapest, Hungary). Immunohistochemistry was conducted with anti-Ki67 antibody and anti-CD31 antibody.

The levels of protein in the mouse skin tissues were measured by ELISA. Mouse skins were homogenized using a Multi-sample Tissue Grinder (Shanghai Jingxin, Tissuelyser-24) following the manufacturer’s instructions. The debris was removed by centrifugation at 13,000 × g for 15 min. The protein levels of TNF-α, β-catenin in the skin tissue extracts were measured using ELISA kits according to the manufacturer’s instructions.

### 5.8 Statistical analysis

All data were presented as the mean ± standard deviation, and each value was averaged or calculated using three unbiased samples collected in the experimental data. Statistical analysis was performed using an independent-sample *t*-test or one-way analysis of variance followed by Tukey’s *post hoc* test on GraphPad Prism software. A statistically significant difference was reported if P < 0.05.

## Data Availability

The original contributions presented in the study are included in the article/Supplementary Material, further inquiries can be directed to the corresponding authors.
